# Developing a peer-led approach to assess the oral health needs and experiences of people in prison and on release

**DOI:** 10.1038/s41405-026-00485-2

**Published:** 2026-09-30

**Authors:** Joelle Booth, Samantha Gould, Robert Witton, Martha Paisi

**Affiliations:** 1 Peninsula Dental School, University of Plymouth, Drake Circus, Plymouth, PL4 8AA UK; 2 Peninsula Dental Social Enterprise, 20 Research Way, Plymouth, PL6 8BT UK; 3 Peninsula Medical School, University of Plymouth, Drake Circus, Plymouth, PL4 8AA UK; 4 School of Nursing and Midwifery, University of Plymouth, Drake Circus, Plymouth, PL4 8AA UK

**Keywords:** Dental epidemiology, Dental public health

## Abstract

**Background:**

This study used a participatory research approach to develop population-specific oral health questionnaires and a peer-researcher model to explore the oral health needs and experiences of people in prison and after their release.

**Methods:**

A two-phase co-design approach using the Double Diamond framework engaged people with lived experience of prison, dental professionals, and support workers. Phase one involved collaborative workshops to design two questionnaires: one for use in prison and one following release. A peer-researcher model was also co-designed. Phase two piloted the questionnaires and peer-researcher model with people who had previously been in prison.

**Results:**

The questionnaire development process indicated that population specific questionnaires, rather than generic oral health questionnaires, were required to capture the distinct experiences of people in prison and following release. This included broader impacts of poor oral health, such as reduced self-esteem, barriers to employment, and substance use to manage dental pain. The peer-researcher model was deemed essential by the engagement group to improve uptake of the questionnaires through building trust. This model also enabled dialogue facilitated by peer-researchers that supported risk reduction (e.g. tobacco use), health education, and signposting to support services.

**Conclusions:**

A co-design approach enabled the development of population-specific oral health questionnaires and a peer-researcher model. These tools can be used in future studies to collect oral health data and support positive changes in both oral and general health behaviours.

## Background

Globally, an estimated 11.5 million people are held in prisons, and in most countries the prison population is increasing [1]. People in prison experience multiple disadvantages such as prior experience of homelessness, substance use, mental ill-health, and higher exposure to trauma and abuse [2–4]. They face significantly poorer mental and physical health compared with the general population [5]. Whilst these inequalities are partly due to higher health needs on entering prison, the prison system itself can contribute to poor health due to structural and environmental barriers [6]. These challenges persist for individuals released from prison, who often face difficulties navigating community healthcare and experience interruptions in care [7].

The health inequalities people in prison face extend to oral health, they have a higher prevalence of untreated dental decay and periodontal disease, and the prevalence of dental infections is five times greater than in the general population [8–10]. People in prison are also more likely to suffer from substance use disorders, which is associated with poor oral health [11, 12]. Poor oral health is associated with pain, embarrassment and difficulties eating, impacting quality of life [13, 14]. On release, evidence suggests that good oral health can play a role in societal reintegration [15].

Oral health questionnaires are a method for assessing the oral health status of a population, capturing self-reported needs, behaviours and impacts [16]. They can evidence inequalities, identify unmet need and inform the design of effective prevention and clinical strategies. Although oral health questionnaires for the general adult population exist, including those used in the Adult Oral Health Survey (AOHS) [17], which incorporates validated tools such as the Oral Health Impact Profile [18], they do not adequately capture the experiences and needs of people in prison. For example, the AOHS asks respondents who have not attended a dental appointment within the past two years to report their reasons for non-attendance; however, the available response options do not capture the structural barriers commonly faced in prison settings [19]. Such barriers include having appointments cancelled due to prison staff shortages. Consequently, utilising existing questionnaires for people in prison and on release risks overlooking key challenges that the criminal justice system places on achieving and maintaining good oral health. People in prison are routinely excluded from national oral health surveys such as the AOHS [17]. This systematic exclusion reflects a lack of prioritisation of this population within routine oral health surveillance and contributes to the underrepresentation of people in prison within population-level oral health data. As a result, oral health inequalities affecting people in prison and on release are likely to be underestimated and insufficiently addressed.

There is a paucity of tangible evidence on the oral health needs and experiences of people in prison. There is even less data that demonstrates the oral health experiences of those who have been released from prison, making it challenging to determine whether they can access appropriate dental services after release and receive continuity of care. One of the reasons for this lack of data is due to the complexities of accessing, recruiting, and engaging people in prison and following their release into the community [6]. This project aimed to address these challenges by adopting a participatory research approach to develop both a questionnaire and an appropriate method of administration. The aim was to develop questionnaires that more accurately reflect the unique oral health needs and experiences of these groups and an administration model that supports recruitment and engagement, considering the logistical and practical constraints of conducting research within prison settings.

## Methods

The project used a participatory approach to develop tailored oral health questionnaires and a peer-researcher administration model for people in prison and following release. This paper describes the co-design process and resulting outputs, rather than generating formal qualitative findings.

This study utilised a co-design methodology to develop questionnaires assessing the oral health needs of people in prison and on release, together with a peer-researcher model for their administration [20]. People with lived experience contributed to the development of the questionnaires and design of the peer-research model. In this paper, ‘peer-researcher model’ refers specifically to questionnaire administration by people with lived experience.

**Fig. 1:**
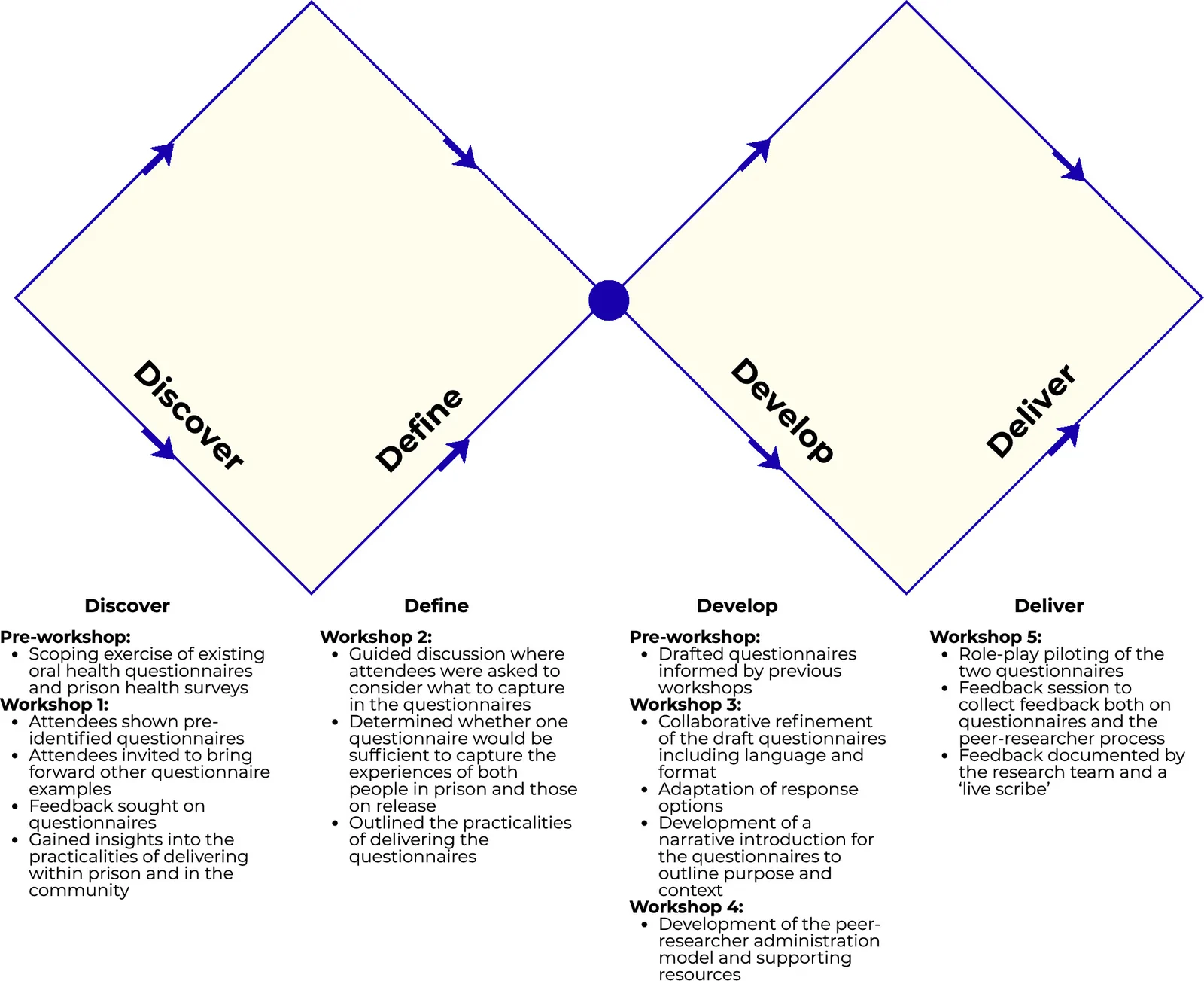
Application of the Double Diamond framework to the co-design process. Overview of how the study activities and workshops were mapped across the four co-design stages (Discover, Define, Develop and Deliver) to develop and pilot the oral health questionnaires and peer-researcher model.

The British Design Council’s Double Diamond framework was used as the co-design approach for this project (Fig. 1) because it provides a systematic way to explore needs and challenges, encourages broad stakeholder engagement, and supports iterative development of solutions [21]. The Double Diamond design process has four steps: 1) Discover, where problems are explored and ideas generated; 2) Define, where problems are refined; 3) Develop, where solutions are created and tested; and 4) Deliver, where the solution is evaluated [22].

### Recruitment

The study was conducted in the Southwest of England. It involved people with lived experience of prison, dental care professionals providing care to people in prison, and staff from third sector organisations that support people both during their time in prison or re-integrating into the community. Ethical approval was obtained from the University of Plymouth Faculty Research Ethics and Integrity Committee (Project ID: 5704). All those involved with the study gave their informed consent to participate.

Several recruitment methods were used to engage multiple stakeholder groups and maximise inclusivity. The opportunity was circulated through existing networks, third sector organisations in the region that support those who have been in contact with the criminal justice system, and through snowball sampling. Participants were remunerated for their time with vouchers in accordance with the National Institute for Health and Care Research guidance [23].

### Workshop overview

**Fig. 2:**
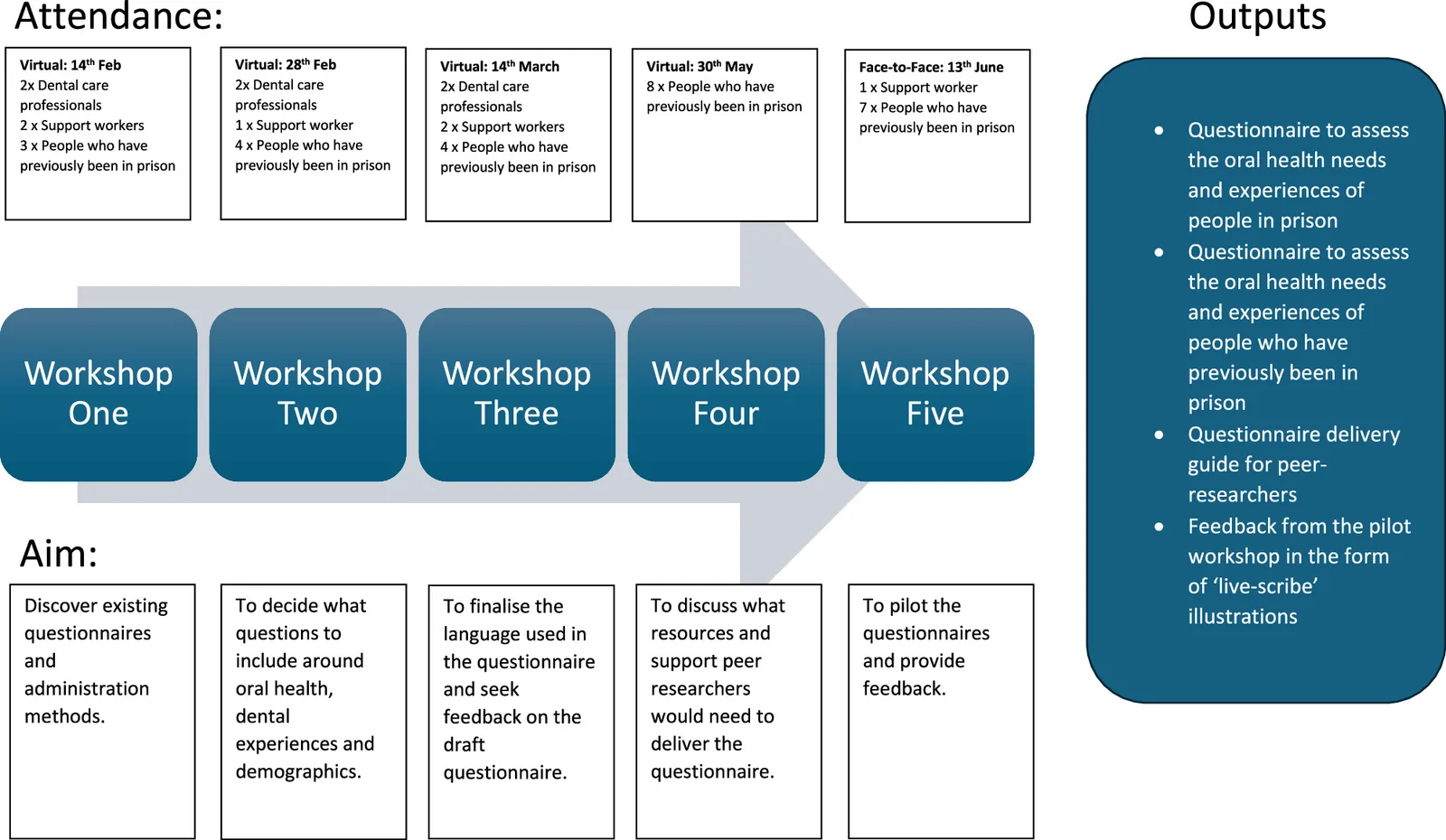
Overview of the co-design workshops. Summary of attendance, aims and outputs mapped across the five workshops used to develop and pilot the oral health questionnaires and peer-researcher model.

The workshops were held between 14^th^ February and 13^th^ June 2025, four of these were conducted online and one in person. An overview of the co-design workshops, attendees, dates and aims can be found in Fig. 2.

### Documentation of workshops

Workshops conducted online were recorded using Microsoft Teams and the auto-transcription feature was used. Following each workshop, two members of the research team produced reflective notes documenting the discussions (either SG or MP and also JB), outcomes, decisions and agreed actions. These notes were subsequently reviewed, combined and updated by one researcher (JB) with reference to the workshop transcripts.

Formal qualitative analysis, such as thematic or framework analysis, was not undertaken. Instead, the workshop records were used to document the co-design process, inform this manuscript and guide iterative changes to the questionnaires and peer-researcher model.

### 1. Discover

The aim of the Discover stage was to explore existing questionnaires and to understand the practicalities of data collection within prisons and community settings.

A member of the academic team (JB) conducted a scoping exercise of existing oral health questionnaires and prison health surveys. Several questionnaires were identified including those used in the AOHS for England and Scotland [17, 24], Public Health England’s Health and Justice Health Needs Assessment Template [25] and the HM Inspectorate of Prisons Prisoner Survey [26].

In the first workshop, the academic team (JB and MP) introduced attendees to key behaviours and wider determinants that can impact oral health, including alcohol use, sugar consumption, substance use, and the ways in which oral health can vary across demographic groups. Attendees then reviewed the identified questionnaires and indicated the suitability of the included questions, format of delivery, question styles and lengths of the questionnaires. Attendees were invited to bring forward other examples they may have encountered through their experience for discussion.

Finally, to gain insights into the practicalities of delivering meaningful questionnaires within prison settings and in the community upon release, workshop attendees were then asked to share their experiences of completing questionnaires in these settings.

### 2. Define

The Define stage aimed to determine the questionnaire content, consider the structure and format of the questionnaires, and clarify whether separate versions were needed for people in prison and those on release.

To address this aim, the second workshop focused on defining the key oral health experiences and needs that the co-design team felt were important to capture and include within the questionnaire. To facilitate these decisions a member of the academic team (JB) asked attendees to consider what would be important to capture within four themes; oral health behaviours, accessing dental services, impacts of oral health and demographics.

Additionally, the workshop considered whether one questionnaire would be sufficient to capture the experiences of both people in prison and those on release, the group concluded that two separate questionnaires would be needed. In addition to this, the group defined the complexities of administrating the questionnaire to these groups that would need to be addressed through the Develop stage. During this stage, the group agreed to adopt a peer-researcher model of administration, recognising that people with lived experience would be best placed to support data collection and enhance engagement.

Decisions were reached through group discussion and consensus. Participants were given the opportunity to review the resulting questionnaire content and peer-researcher model at the subsequent workshop, with further amendments made where required.

### 3. Develop

The Develop stage aimed to create and iteratively refine draft questionnaires and supporting materials. This stage also aimed to develop a peer-researcher model that could be used to deliver the questionnaires.

Before workshop three, one of the researchers (JB) produced draft questionnaires informed by feedback from workshop one and by the oral health experiences identified in workshop two. During the workshop, the group collaboratively refined the draft questionnaires, focusing on language, clarity and the preferred question formats (e.g., tick boxes, rating scales, pictorial options). Further adaptations were made to ensure the response options were appropriate and relevant for people currently in prison and for those on release, tailoring the questionnaire to each group. Within this workshop, a narrative introduction was developed to include at the beginning of the questionnaires that explains the purpose and context. Final questionnaire items were selected through group consensus, considering their importance to participants, clarity, acceptability and the need to maintain an appropriate questionnaire length.

The fourth workshop was attended only by those with lived experience of previously being in prison. This workshop focused on developing a peer-researcher model of administration for the questionnaires and accompanying resources to support this. To develop the model of administration the group were asked to consider the following: who should deliver the questionnaire, what resources and support would be needed by someone delivering the questionnaire, and how participants could be recruited.

### 4. Deliver

The Deliver phase aimed to pilot and test the developed questionnaires and administration approach through simulated role-play exercises, gathering feedback from participants. This stage aimed to finalise the questionnaires and administration approach prior to future testing in prison and community settings.

In the final workshop, which was conducted in person, attendees were given an overview of both the questionnaires and the peer-researcher approach. All but one participant had attended at least one previous workshop.

Participants were then divided into pairs, with one person acting as the peer-researcher and the other as a questionnaire responder. This enabled the questionnaire to be piloted through a role-play scenario. The exercise was repeated so that participants could switch roles (peer-researcher or respondent), administration method (paper or digital), and questionnaire type (people in prison or people on release). During the role-play activities, each pair was observed by a member of the research team or a support worker, who aided, answered questions, and recorded feedback.

**Fig. 3:**
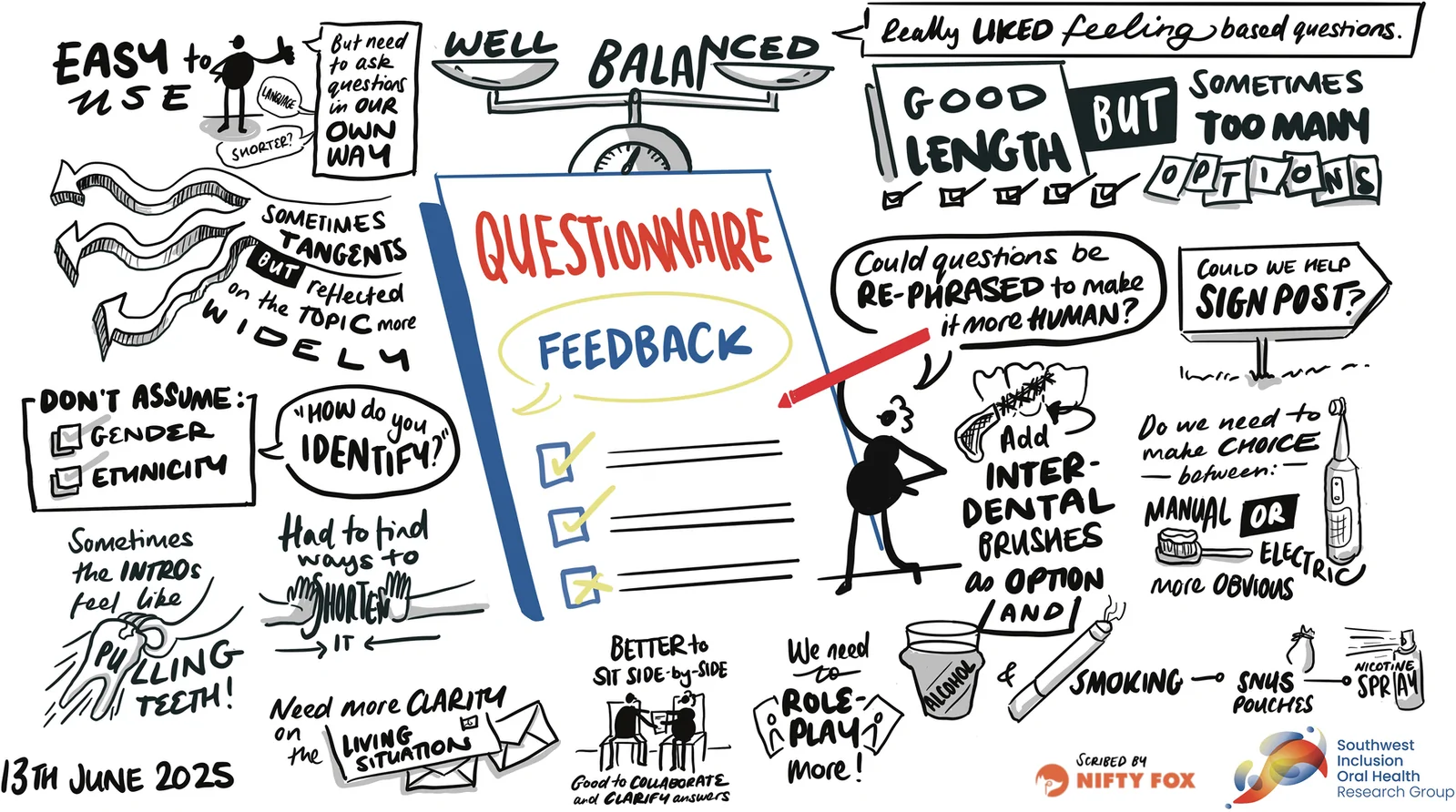
Live-scribed feedback on the oral health questionnaires. Visual summary of feedback from the final co-design workshop, during which participants piloted the prison and post-release questionnaires through role-play. Feedback focused on questionnaire length, language, response options and the value of side-by-side administration by peer-researchers.

**Fig. 4:**
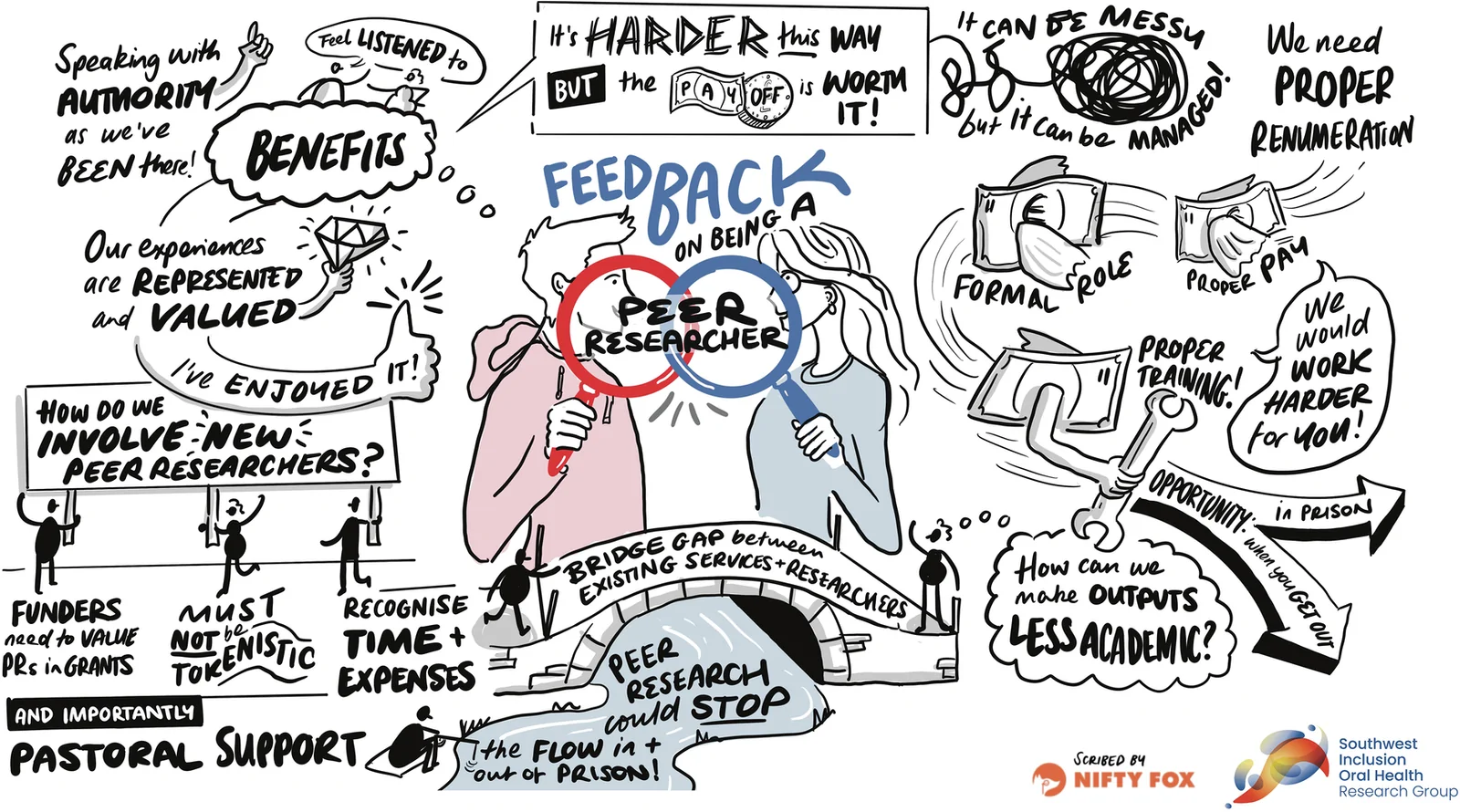
Live-scribed feedback on the peer-researcher model. Visual summary of feedback from the final co-design workshop. Participants reflected on the benefits of lived experience, trust and peer engagement, alongside the need for training, remuneration, pastoral support and meaningful involvement.

Following the role-playing activity, the second half of the session was dedicated to seeking feedback on both the questionnaires and peer-researcher model. The feedback was documented by the research team and a ‘live scribe’ who joined the session virtually, capturing outputs from the discussion in real time. Feedback from the role-play activities was subsequently reviewed by a member of the research team (JB) and incorporated into the final questionnaires and peer-researcher model. The live-scribe outputs presented in Figs. 3 and 4 were used to inform these refinements.

## Results

The process included 13 individuals: eight people who had experience of being in prison, two dental professionals who deliver care in prisons, two people who support people in prison and on release, and one individual who had experience of being in prison and now works as a support worker. Of the 13 individuals involved, six were female. Further demographic and custodial-history information, including age, ethnicity, length of prison experience and types of prisons represented, was not collected.

Through the co-design process two questionnaires were developed, one for people in prison (S1) and one for people on release (S2). Alongside each questionnaire, a peer-researcher guide was developed to support administration of the questionnaires (S3–4). The principal questionnaire domains are summarised in Table 1. The two questionnaires covered common domains but included context-specific questions and responses relating to experiences in prison and following release. A lay audience overview of the project can be accessed in the supplemental material (S5).

**Table 1:** Summary of domains included in the oral health questionnaires.

Domain	Illustrative Content
Oral health related behaviours	Oral hygiene practices, sugary food and drink consumption, alcohol use, smoking, vaping, smokeless tobacco, nicotine replacement products and support for substance use.
Dental attendance and service use	Timing and reasons for previous dental attendance, dental care received while in prison, and use of other healthcare services for dental problems, including accident and emergency departments, general practice and prison healthcare services.
Access to dental care and continuity of care	Awareness of how to access care, waiting lists, cancelled appointments, prison transfers and interrupted treatment, prison staffing constraints, and, following release, availability of NHS dental care, affordability and transport.
Oral health symptoms and wider impacts of oral health	Dental pain, difficulty eating, dental anxiety, embarrassment, confidence, social interactions, employment and use of substances to manage dental pain.
Demographic and contextual information	Age, gender and ethnicity, alongside current prison type or post-release living and housing circumstances.
Additional experiences	An open-text opportunity for respondents to describe other relevant oral health needs or experiences.

Questions and response options were adapted to reflect the different circumstances experienced in prison and following release. The complete questionnaires are provided in Supplementary Materials S1 and S2.

### Discover and define stages

#### Questionnaire development

When participants reviewed existing questionnaires, such as the AOHS [17], these were consistently thought to be too long, overly complex and inaccessible for people in prison and on release who may struggle with literacy. Those with lived experience of being in prison emphasised that lengthy or complex questionnaires were likely to reduce engagement. They also highlighted that extensive questions relating to demographics, housing or income could feel intrusive and deter these populations from engaging due to their mistrust of those in authority. To address this, participants stressed the importance of transparency about the questionnaire’s purpose, concise and accessible wording, and inclusion of only essential questions. The group felt it was essential to explain that completing the questionnaire would not improve or restrict access to dental care.

In relation to the questionnaire content, first the academic team presented an initial set of topics relevant to understanding oral health. The wider stakeholder group then discussed, refined, and agreed on the final list of topics to include. The group identified several key areas they felt should be captured: oral health behaviours, wider health behaviours such as substance use and tobacco use, access to dental care in both prison and community settings, and the broader personal impacts of poor oral health. These wider impacts included pain, reduced self-confidence, difficulties with employment, and reliance on substances to self-manage dental pain. Participants also noted that poor oral health can influence behaviour, affecting day-to-day interactions and overall wellbeing. They further highlighted that limited access to routine dental services following release from prison may lead individuals experiencing dental pain to seek care through accident and emergency departments, which should therefore be included.

#### Peer-researcher model

Discussions also highlighted the need for an administration model for the questionnaire that considers the structural and literacy barriers commonly experienced by people in prison and on release. Participants advocated for face-to-face administration of the questionnaire through peer-researchers. It was felt that peer-researchers could facilitate trust and engagement, walking responders through the questionnaire, explaining questions in accessible language and supporting those who might struggle with comprehension. Within the prison environment, it was also felt that peer-researchers could overcome some of the logistical complexities of conducting research in prisons. For example, they could support recruitment by embedding the questionnaire within existing peer mentor models, such as those used in health-related interventions.

The Discover and Define stage findings outlined the need for a short, inclusive questionnaire that was transparent about what impact partaking in the questionnaire would have. The questionnaire needed to capture both the structural barriers experienced relating to accessing dental care in prisons and in the community but also the impacts of poor oral health on wider health and wellbeing. A peer-researcher, face-to-face administration model was perceived by participants to be acceptable and potentially feasible, particularly within prison settings, and it was felt that this approach could enhance data quality and participation among these populations.

#### Develop and deliver stages

Participants emphasised the need for person-first, non-stigmatising language, the use of plain English, and trauma-informed phrasing throughout the questionnaire. The terms ‘people in prison’ and ‘people who have previously been in prison’ were preferred by the group.

Structural recommendations were also made such as including a maximum length of 15–20 questions and having a mix of multiple-choice and short-answer formats. To support questionnaire completion and prevent feelings of mistrust, participants recommended that the questions should be ordered to start with the least sensitive questions and ending with the more intrusive questions. The group emphasised the need to make clear to respondents that “prefer not to say” was an available option for every question, and that peer-researchers should be prepared to reiterate this choice whenever needed.

The questionnaire was adapted by the group to include further responses to capture the practical challenges experienced when trying to access dental care both in prison and on release, these issues included relying on emergency departments for the management of dental pain, being unaware of how to access dental care and systemic gaps that could mean that they were unable to access dental care in prison such as prison staff shortages.

Participants felt that those undertaking the peer-researcher role would benefit from a guide to support questionnaire administration. The questionnaire guide was developed to include information on why certain questions were included in the questionnaire such as outlining that the reason the questionnaire asks about a participant’s demographic and topics that may feel are intrusive such as substance use.

Further questionnaire feedback was captured after piloting the two questionnaires in role-playing scenarios through the ‘live scribe’ (Figs. 3 and 4). The piloting highlighted the need to further refine the questionnaire, such as including options to capture the use of nicotine replacement therapies and snus when asking about tobacco and smoking histories. Peer-researchers reported that the questionnaires were straightforward and appropriately worded yet found that in practice they delivered them in their own individual ways, developing their own personal styles. They found that administering the questionnaires worked best when sat side-by-side with the respondent, allowing for opportunities to clarify answers and collaborate.

The administration of the questionnaires facilitated wider dialogue about oral and general health, with peer-researchers noting that this process enabled them to provide health messaging and direct respondents to support services for health behaviours such as tobacco control. Peer-researchers reflected that were they to deliver the questionnaires in a real-world context, they would value oral health education training to equip them with the knowledge to offer appropriate oral health advice and to signpost respondents to suitable support services.

Feedback also highlighted the perceived value of the peer-researcher role. Participants felt that peer-researchers brought a unique authenticity to the process because of their lived experience, enabling them to connect more effectively with respondents. They were viewed as essential in bridging the gap between existing services, academic teams, and the people who use those services. The peer-researcher model was described as a potential mechanism for disrupting the ‘revolving door’ of repeated prison sentences. Individuals with lived experience explained that developing transferable research skills whilst in prison could enhance self-worth, offer a sense of purpose, and create opportunities for employment upon release.

The group also reflected on the co-design process more broadly. They acknowledged that co-design can feel “messy” and is often a more demanding way to conduct research, but emphasised that the benefits outweigh these challenges because it genuinely incorporates, values, and represents lived experience. Through their involvement, peer-researchers felt that the questionnaires were more likely to reflect the real needs, priorities, and circumstances of people in prison and those preparing for release, making the questionnaires and data captured through them more relevant, acceptable, and ultimately more effective.

To sustain meaningful participatory research partnerships, participants stressed that systems must support this way of working through appropriate remuneration, ongoing support, and a commitment to ensuring that those involved are genuinely heard. They highlighted the importance of avoiding tokenism and creating conditions in which lived experience can meaningfully shape both the research process and its outcomes.

## Discussion

### Summary of key findings and links to wider literature

This co-design study led to the creation of two newly developed questionnaires to capture the oral health needs and experiences of people in prison and on release. These questionnaires can be used in future studies to capture the unique experiences of these groups such as structural barriers to accessing dental care and the broader impacts of poor oral health such as a barrier to employment and societal integration. The study also demonstrated the benefits of delivering the questionnaires through a peer-researcher model, such as supporting a trauma-informed and accessible approach whilst addressing literacy barriers and mistrust that often impact these groups.

A key concept is that peer-researcher administration of the questionnaires extends beyond the transactional collection of data. It creates a platform for meaningful conversations about health and oral health behaviours and facilitates signposting to relevant support services. This theme was consistently highlighted during the questionnaire development workshops and reinforced throughout the role-play scenarios. Peer-researchers reported that the questions prompted discussions, allowing them to share knowledge about oral health behaviours and signpost participants to appropriate support when needed. This could aid the improvement of oral health behaviours as qualitative research has indicated that people in prison are sceptical of advice given by those in authority and are more likely to base their health-related choices on advice from those they trust, such as peer-researchers [27].

Throughout this work, the benefits for those involved as peer-researchers was stressed. The group discussed how being a peer-researcher could improve self-confidence, be an opportunity to learn new skills, lead to employment opportunities, provide purpose whilst in prison and help them to feel part of the community on release. They suggested that these could prevent the ‘revolving door’ cycle whereby individuals repeatedly cycle in and out of prison. A small study conducted in Scotland focused on training people in prison as health coaches as part of an oral health intervention evidenced similar benefits through improved self-esteem, self-efficacy, improvements in mood and health behaviours such as toothbrushing and physical activity [28, 29]. This suggests that using a peer-researcher model could contribute to rehabilitation, reducing reoffending and supporting future employment.

Peer-researchers have been successfully used within justice settings such as to explore the impact of lockdowns on people in prison due to the COVID-19 pandemic [30]. The study used a similar approach whereby academics worked alongside those with lived experience of the justice system to design a mixed-method study, a component of which used a prison-based peer-delivered survey. The study demonstrated that a peer-researcher model was feasible with 60 prisoners trained as peer-researchers across 11 prisons, resulting in 1421 surveys being completed. Peer-researchers can build rapport, explain research in accessible language, and navigate the practical challenges of prison routines, enhancing recruitment, engagement and data collection [31–33]. However, peer-researcher models may also present challenges. Maintaining research quality requires structured training, clear administration guidance and ongoing supervision, while variation in how peer-researchers explain or deliver questions may introduce bias. Longer-term sustainability will also depend on appropriate remuneration and support systems.

A recurring theme for discussion throughout this study, was an emphasis on how those who have lived experience of the justice system are reluctant to engage with research that does not lead to tangible action. Participants were passionate about having their voices heard but wanted clarity on how their involvement would contribute to change and where the findings would be shared. These findings echo previous studies in justice settings and emphasises the importance of clearly articulating the purpose, expected impact, and dissemination plans when inviting participants to engage in research [34].

### Strengths and limitations

The principal strength of this study lies in its participatory approach, which positioned people with lived experience of prison at the centre of the co-design process. In justice and health research contexts, co-design has been shown to enhance the relevance, credibility, and acceptability of research outputs [35–38]. This approach increases the likelihood that both the questionnaires and administration method will be feasible, acceptable, and effective, particularly because it engaged people from diverse backgrounds who contributed a range of insights.

This study has several limitations. The co-design process, guided by the Double Diamond framework, is inherently iterative, meaning that the questionnaires and peer-researcher model developed here should be considered evolving tools rather than fixed instruments [21]. Further refinement may be required as they are implemented in different prison or community settings, and additional co-design cycles may be necessary to ensure they remain relevant, accessible, and responsive to the needs of people in prison and those on release.

Additionally, the sample was relatively small and recruited from one region of England, the Southwest, which may limit the transferability of the findings. Limited demographic and prison history information was collected, which further restricts assessment of the representativeness of the participant group. Given the small sample and potential risk of identification, only limited participant characteristics have been reported. Participants who chose to take part may also have been particularly motivated to engage with research or interested in oral health, which may have introduced selection bias. In addition, only two dental professionals participated, which may have limited the range of professional perspectives represented. Although people with lived experience of prison were central to the co-design process, no participants were currently in prison, which may have limited the extent to which the process captured current prison conditions and constraints, and therefore the transferability of the findings to contemporary prison settings.

The questionnaires and peer-researcher model were piloted through simulated role-play rather than administered in prison or post-release community settings, and all but one participant in the role-play exercise had contributed to at least one earlier co-design workshop. The findings therefore indicate perceived acceptability and potential feasibility but do not establish the validity or reliability of the questionnaires, or the feasibility of real-world implementation within the intended populations and settings.

### Future directions

Future research should pilot the questionnaires and peer-researcher model in prison and community settings with people who were not involved in their development. This should assess the feasibility and acceptability of administration among respondents, peer-researchers and service providers. It should also evaluate face and content validity, reliability and construct validity among both people in prison and people following release. Successful implementation will require adequate resources, including training for peer-researchers in oral health education, research ethics, and questionnaire administration, as well as ongoing support and supervision to ensure consistency and quality of data collection.

## Conclusion

This study demonstrates the value of a co-designed peer-researcher model for assessing the oral health needs of people in prison and on release. The tailored questionnaires and administration model reflect lived experience, and during simulated role-play, supported engagement and meaningful conversations about oral health behaviours and access to support. With further testing in prison and community settings, alongside appropriate training and supervision, this approach offers a promising method for capturing data to inform action to reduce oral health inequalities in these underserved populations.

## Supplementary information

S1 Questionnaire - UNDERSTANDING THE DENTAL NEEDS AND EXPERIENCES OF PEOPLE IN PRISON

S2 Questionnaire - UNDERSTANDING THE DENTAL NEEDS AND EXPERIENCES OF PEOPLE WHO HAVE PREVIOUSLY BEEN IN PRISON

S3 Questionnaire Guide - UNDERSTANDING THE DENTAL NEEDS AND EXPERIENCES OF PEOPLE IN PRISON

S4 Questionnaire Guide - UNDERSTANDING THE DENTAL NEEDS AND EXPERIENCES OF PEOPLE WHO HAVE PREVIOUSLY BEEN IN PRISON

S5 Peers with Purpose One Page Infographic Summary. Developed by NiftyFox

## Data Availability

The datasets used and/or analysed during the current study are available from the corresponding author on reasonable request. Materials are available within the supplemental documents.
