# Supplementary material for: Developing a peer-led approach to assess the oral health needs and experiences of people in prison and on release
Source: BDJ Open. 2026 Sep 30;12:93. doi: 10.1038/s41405-026-00485-2 (PMC13627706; doi:10.1038/s41405-026-00485-2)
Supplement: Supplementary file 1 [file 41405_2026_485_MOESM1_ESM.pdf]

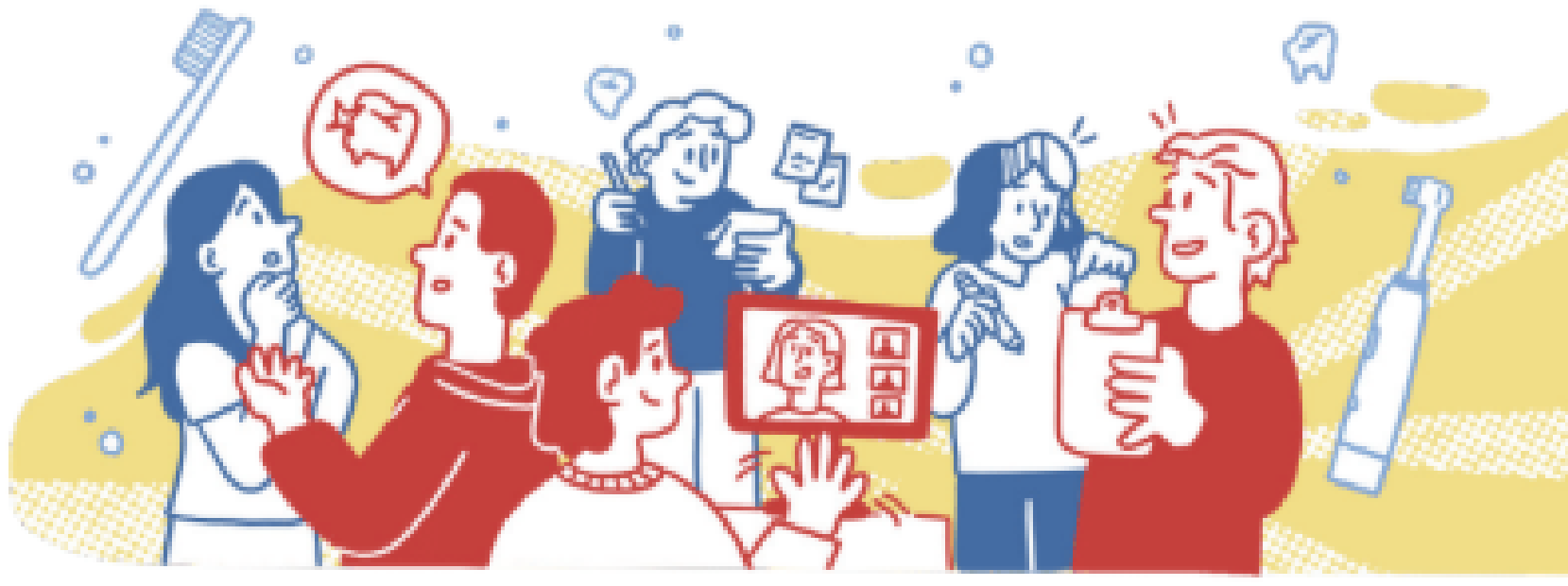

UNDERSTANDING THE DENTAL NEEDS AND EXPERIENCES OF PEOPLE IN PRISON

We would like to ask you some questions about you and your dental health. This questionnaire has been created with people who have experience of being in prison, people who support those in prison and prison dental teams. The questionnaire will take around 10 minutes to complete.

The questions are designed to help us to understand the dental needs of people in prison and help us design dental services and programmes to improve their dental health.

The answers you give in this questionnaire will be anonymous and will not be shared with authorities. None of the answers you give will affect any of the care or support you currently receive or your ability to access care in the future. Filling out this questionnaire will not directly give you access to a dentist but may facilitate more access to dentistry for people in prison in the future and help to improve their dental health.

If you do not feel comfortable answering any of these questions, please leave them blank.

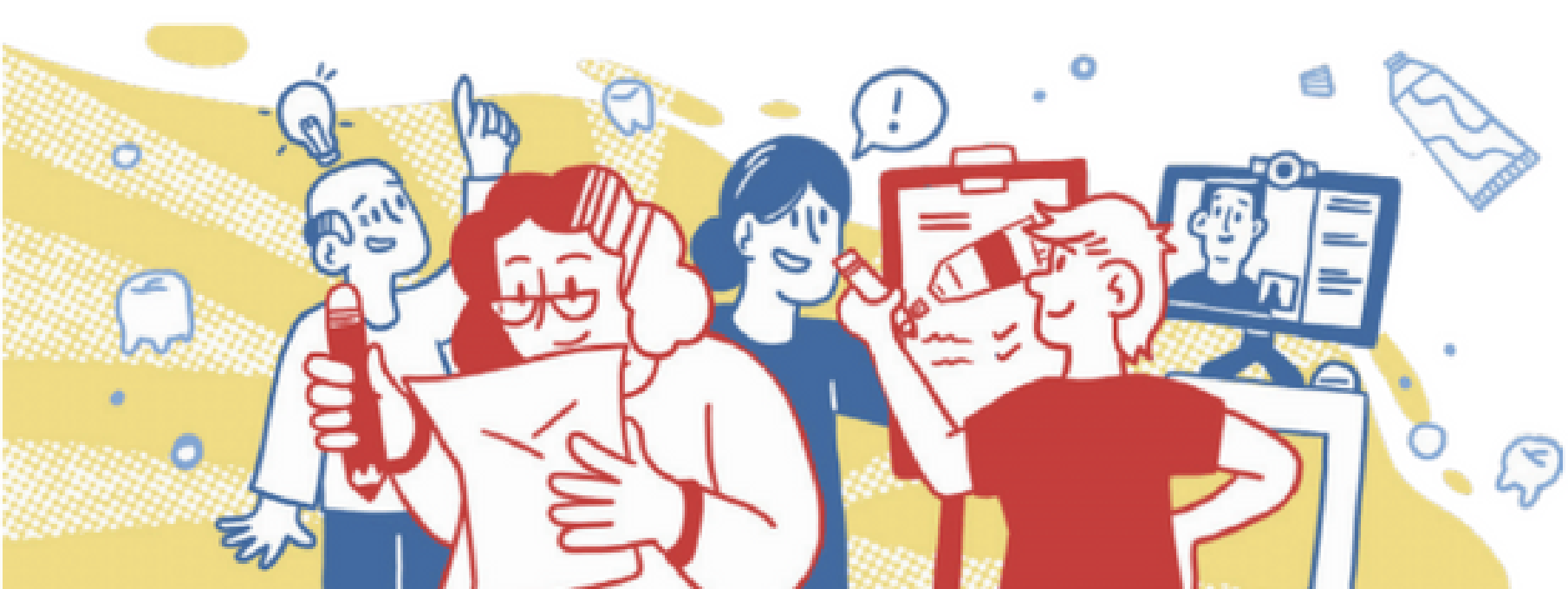

Thank you for your time!

Looking after your teeth:

Which of these do you use to clean your teeth or dentures and how often do you use them?

	Less than once a week	Less than once a day	Once a day	Twice a day	More than twice a day
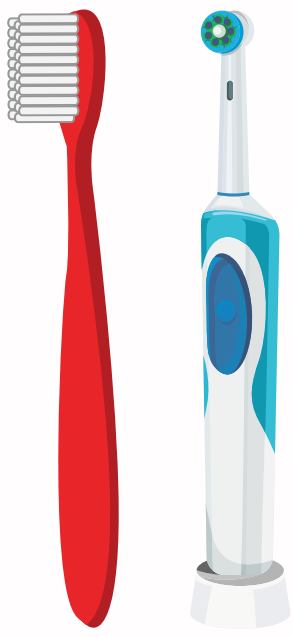 Toothbrush					
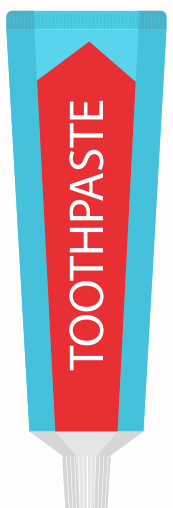 Toothpaste					
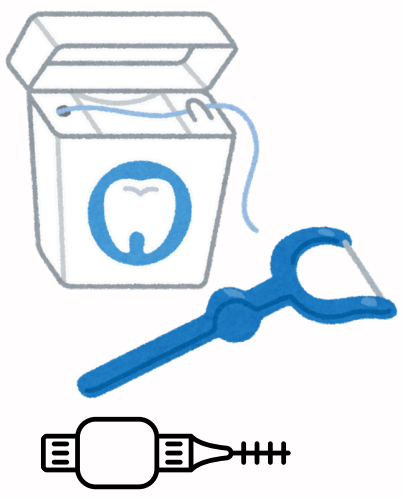 Dental floss/ Interdental brushes					
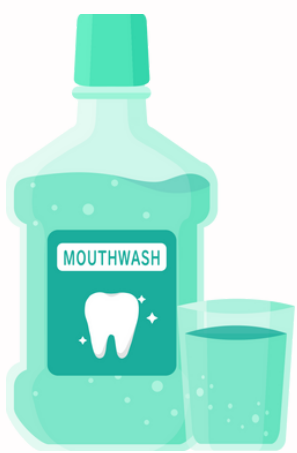 Mouthwash					
Other (*please write*)					

How long ago was your last visit to the dentist?

- ◯ Never or not since childhood
- ◯ Within the last year
- ◯ Between 1 to 2 years ago
- ◯ Between 3 to 10 years ago
- ◯ Over 10 years ago

Why did you last visit a dentist?

- ◯ Due to pain in my mouth
- ◯ Due to an abscess or swelling
- ◯ For a check-up
- ◯ I needed to see the dentist but was unable to
- ◯ For dental treatment such as filling or to have dentures made
- ◯ I can't remember

Other *(please write)*

If you have not visited a dentist recently:
Which of these reasons, if any, are why you have not been to the dentist?
(Tick all that apply)

- ◯ I have not tried to see a dentist
- ◯ I don't know how to access the dentist
- ◯ I am on the waiting list to see the dentist but have not yet been seen
- ◯ I was transferred to a new prison before I reached the top of the waiting list to see a dentist
- ◯ I was transferred to a new prison whilst I was part way through my treatment
- ◯ The prison I am in only offers appointments for dental emergencies
- ◯ My appointment has been cancelled due to a prison lockdown
- ◯ My appointment has been cancelled due to there being no prison officers to escort me to the appointment
- ◯ My appointment has been cancelled due to there being no prison dental staff available
- ◯ I feel too embarrassed to go to the dentist
- ◯ I had a bad experience at the dentist
- ◯ I am afraid or anxious of going to the dentist
- ◯ I attended accident and emergency (A&E) for a dental problem as I could not see a dentist
- ◯ I saw one of the prison doctors or nurses for a dental problem as I could not see a dentist
- ◯ Other things in my life are more important at the moment
- ◯ I have not had toothache or been in pain

Other *(please write)*

If you went to the dentist for treatment tomorrow, how would you feel?

Not anxious	Slightly anxious	Fairly anxious	Very anxious	Extremely anxious
○	○	○	○	○

Have you seen a dentist whilst you have been in prison?

- ○ Yes
- ○ No. I have tried to see a dentist but have unable to
- ○ No. I have not tried to see a dentist

If yes, how many times have you seen a dentist whilst you have been in prison?

- ○ Once or twice
- ○ Multiple times

If yes, why have you visited a dentist whilst you have been in prison?

- ○ I was in pain or had a dental emergency
- ○ For a course of dental treatment or check-up

How often during the last year...	Never	Occasionally	Very often
... have you had **toothache or painful gums?**			
... have you found it **uncomfortable to eat any foods** because of problems with your teeth, mouth or dentures?			
...have you been **self-conscious, embarrassed or ashamed** because of your teeth, mouth or dentures?			
...felt that your teeth have affected your ability to **get a job**?			
...felt that your teeth have affected your **confidence and the way you interact with others**?			
...have you needed to **use drugs or alcohol to manage dental pain?**			
...felt that your teeth have caused you to **act differently?**			

Sugar can cause dental decay (holes in our teeth).

Which of these best describes how often you have sugary drinks and foods such as energy drinks, chocolate, sugar in tea or coffee, soft drinks etc:

- ◯ Less than once a day
- ◯ Between 2 to 4 times a day
- ◯ More than 4 times a day

Do you normally have these:

- ◯ With meals
- ◯ Between meals
- ◯ Both with and between meals

Alcohol, smoking and substance misuse can affect the health of our teeth and mouth.

Are you currently or have you ever received support for the following? (*Tick all that apply*)

- ◯ Smoking
- ◯ Alcohol
- ◯ Substance misuse
- ◯ I'd prefer not to say

How often did you used to have a drink containing alcohol?

- ◯ Daily
- ◯ 2-3 times a week
- ◯ Monthly
- ◯ 4-6 times a week
- ◯ I'd prefer not to say
- ◯ I did not drink alcohol

How many drinks containing alcohol did you used to have on a typical day when you were drinking?

- ◯ 10 or more
- ◯ 5 to 9
- ◯ 1 to 4
- ◯ I did not drink alcohol
- ◯ I'd prefer not to say

If you did drink alcohol, what types of drinks did you usually have?

- ◯ Beer
- ◯ Cider
- ◯ Wine
- ◯ Spirits (e.g. vodka, whisky, gin, rum)
- ◯ Other, *please write below*
- ◯ I'd prefer not to say

Which of these best describes your experience of smoking and tobacco? (*Tick all that apply*)

- ◯ I currently smoke tobacco
- ◯ I used to smoke tobacco
- ◯ I have never smoked
- ◯ I'd prefer not to say
- ◯ I currently vape
- ◯ I used to vape
- ◯ I have never vaped
- ◯ I use smokeless tobacco such as snus or snuff
- ◯ I use nicotine replacements such as a spray, patches, gum

What gender do you identify as?

- ◯ Male
- ◯ Female
- ◯ Non-binary
- ◯ Other, *please write below*
- ◯ Prefer not to say

What is your age?

- ◯ 16 - 18 years
- ◯ 19 - 29 years
- ◯ 30 - 39 years
- ◯ 40 - 49 years
- ◯ 50 - 59 years
- ◯ 60 - 69 years
- ◯ 70 or over
- ◯ I'd prefer not say

How would you describe your ethnicity?

Which of these best describes your living situation?

- ◯ Category A prison
- ◯ Category B prison
- ◯ Category C prison
- ◯ Category D prison
- ◯ Remand prison
- ◯ Young offender institution (YOI)
- ◯ Youth custody
- ◯ Immigration removal centre
- ◯ Secure children's home
- ◯ Secure training centre
- ◯ Other, *please write below*
- ◯ I'd prefer not to say

Anything else you would like to tell us?

Thank you for completing the questionnaire, the answers you provide will help to give information on the dental experiences of people in prison and help us to improve their dental health.

Southwest Inclusion Oral Health Research Group
