# Supplementary material for: Developing a peer-led approach to assess the oral health needs and experiences of people in prison and on release
Source: BDJ Open. 2026 Sep 30;12:93. doi: 10.1038/s41405-026-00485-2 (PMC13627706; doi:10.1038/s41405-026-00485-2)
Supplement: Supplementary file 4 [file 41405_2026_485_MOESM4_ESM.pdf]

QUESTIONNAIRE GUIDE

UNDERSTANDING THE DENTAL NEEDS AND EXPERIENCES OF PEOPLE WHO HAVE PREVIOUSLY BEEN IN PRISON

Thank you for being a peer researcher for this project.

This questionnaire has been created with people who have experience of being in prison, people who support those in prison and prison dental teams.

Those who created the questionnaire felt strongly that the questionnaires should be delivered by 'peers', like yourself, who have lived experience of being in prison and can help people to fill out the questionnaire.

This guide will help you to deliver the questionnaire.

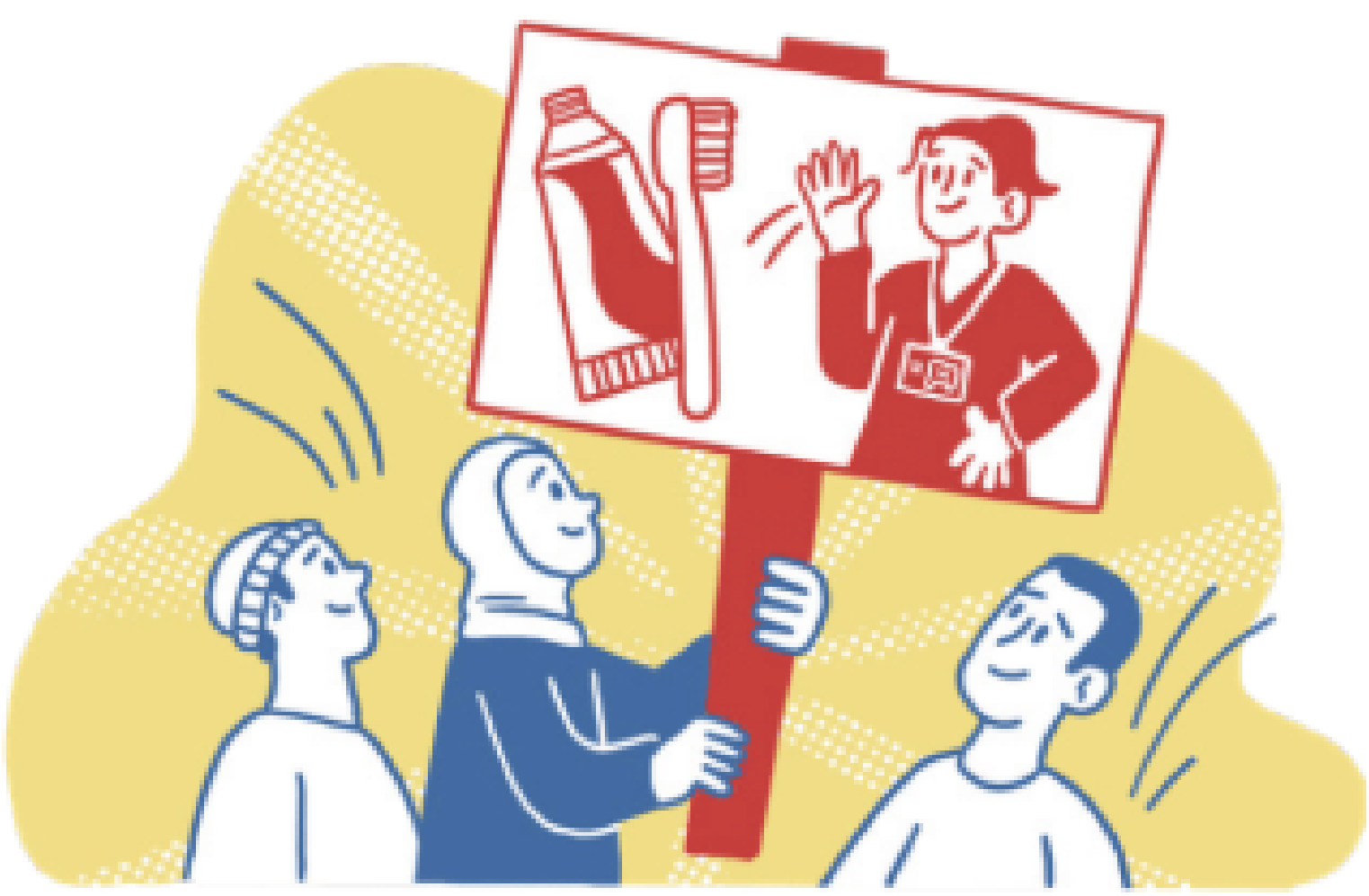

The questionnaire process:

Step 1: Introduce yourself and explain that you are a peer researcher. Check that the person you are completing the questionnaire with has not completed the questionnaire before.

Step 2: Tell the person the information on the front of the questionnaire. With time you may wish to find your own words to explain this information or you may choose to read the section written. The main things to tell the person are:

- Why you are asking them the questions
- That their answers will not give them access to a dentist
- The their answers will not affect any of the care or support they receive and will not be shared with authorities
- They do not need to answer any questions they don’t feel comfortable answering

Step 3: Support the individual to complete the questionnaire

Step 4: Thank the person who has completed the questionnaire for their time

Step 5: Return the completed questionnaire to the research team

Who can fill out the questionnaire?

This questionnaire can be filled out by anyone who has previously been in prison who is over the age of 16. It does not matter whether they have recently seen a dentist in or visited a dentist in prison.

Why fill out the questionnaire?

People may ask you why they should fill out the questionnaire and what it is being used for.

This questionnaire is being used to capture information on the teeth and dental experiences of people who have previously been in prison. This is important because without this information it is difficult to know what improvements need to be made in dental services and how to improve the dental health of people who have been in prison. .

Everyone has a choice as to whether they fill out the questionnaire.

It is important that they know that none of the answers they give will affect any of the care or support they currently receive or their ability to access care in the future. Filling out this questionnaire will not directly give them access to a dentist.

What can I do if someone is struggling to pick an answer?

Sometimes people may struggle to choose an answer. Please help them to choose the answer that best fits them or their experience if there is not an option to select ‘other’. If people feel uncomfortable answering a question please leave this blank or select ‘I’d prefer not to say’.

This section will help you answer any questions you might get about the questionnaire

Why do we ask about sugar?

This is because we know sugar causes dental decay (holes in our teeth). Sugar between meals has a greater risk of causing dental decay then if we have it with meals. This is why we have a question about when people eat or drink sugar.

Why do we ask about substance use?

Certain substances such as heroin, cocaine, cannabis and ecstasy can affect our teeth and gums. They can cause us to grind our teeth, cause dental decay or gum disease. Methadone (given to help people stop using heroin) that contains sugar can also cause dental decay.

Why do we ask about alcohol?

Alcohol can cause problems with our teeth and mouth. Alcohol can contain sugar that causes dental decay. Alcohol is also linked to mouth cancer and can increase the risk of accidental damage to our teeth or face.

Why do we ask about smoking?

Smoking can cause problems with our teeth and mouth. Smoking can cause gum disease and increase our risk of mouth cancer.

Why do we ask about age, gender and ethnicity?

We ask about peoples age, gender and ethnicity as these can affect the health of our teeth and mouth. Our characteristics can affect how easy it is for us to look after our teeth and see a dentist. This information also allows us to compare the health of your teeth and dental experiences with people of a similar age, gender and ethnicity who have not been in prison.

It is important that you ask the person completing the questionnaire what their age, gender and ethnicity is and do not make assumptions or answer this for them.

Why do we ask about living situations?

We ask about peoples living situation as this can affect how easy it is for them to see a dentist.
