# Supplementary material for: Developing a peer-led approach to assess the oral health needs and experiences of people in prison and on release
Source: BDJ Open. 2026 Sep 30;12:93. doi: 10.1038/s41405-026-00485-2 (PMC13627706; doi:10.1038/s41405-026-00485-2)
Supplement: Supplementary file 5 [file 41405_2026_485_MOESM5_ESM.pdf]

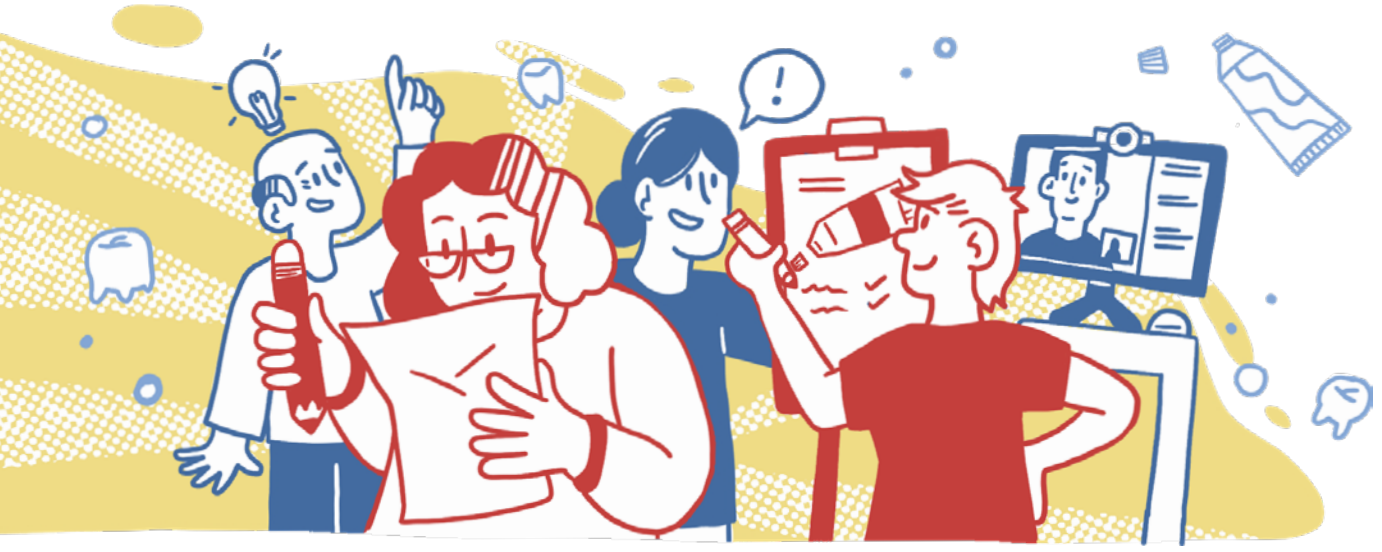

Peers with Purpose:

Using a Peer Research Approach to Measure Dental Health in Prison and Beyond

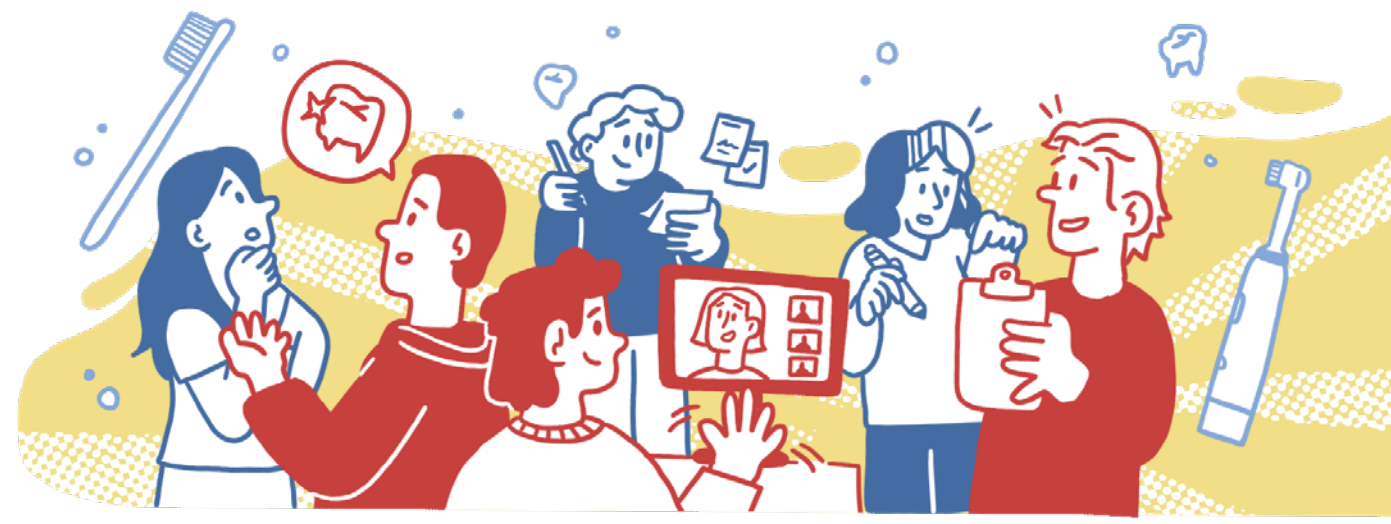

Why Dental Health Matters

For people in prison and on release, dental pain, broken teeth, or missing teeth are more than just health issues. They can affect confidence, relationships, behaviour, job opportunities, and employment. Poor dental health can also lead to isolation, drug or alcohol use to cope with pain, and barriers to starting over.

The Evidence Gap

We know dental health is a serious problem, but data is scarce. Existing surveys don't reflect prison life or the challenges of release, making service improvements harder.

Our Approach

We worked with dentists, prison health staff, support workers, and people with lived experience of prison. Across four online workshops, we:

- Adapted surveys and added new questions to reflect real experiences.
- Created a peer-researcher model, where people with prison experience lead the research.

We then tested the approach in person, role-playing and refining each step.

References:

Booth J, McMullen H, Rodriguez A,Muirhead V. Co-designing a film showcasing the dental experiences of community returners (ex-offenders). Front Oral Health. 2025 Jan 6;5:1391438.

Booth J, O'Malley L, Meek R, Goldrick NM, Maycock M, Clarkson J, Wanyonyi-Kay K. A scoping review of interventions to improve oral health in prison settings. Community Dent Oral Epidemiol. 2023 Jun;51(3):373-379

Key Findings

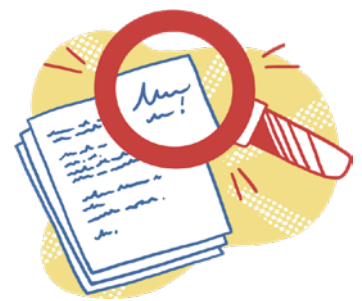

Tailored surveys capture key impacts - such as low self-esteem, work barriers, and coping with pain.

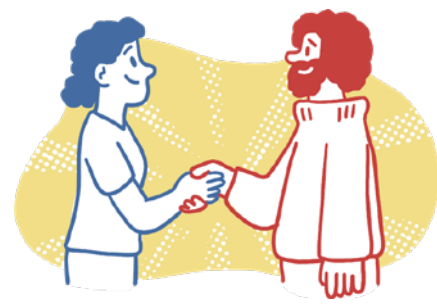

Peer-researchers improve trust, engagement, and response rates. The role also offers employment for people with lived experience.

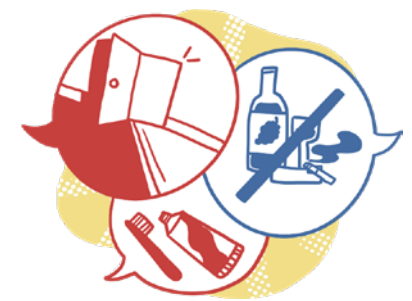

Peer-led conversations go beyond surveys - opening discussions on dental care, access, and wider support.

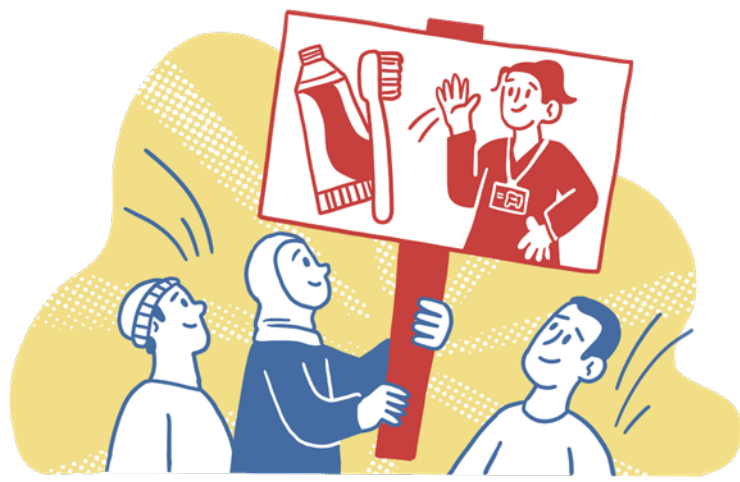

What now?

Dental health must be a clear priority in prisons and after release.

Our peer-led model and questionnaire collects the evidence needed to advocate for better services. Peer-researchers don't just gather data - they build trust, start conversations, and bring community benefit.

Take Action

Do you work in prisons or with people after release? Use our peer-led survey to gather evidence and drive change.

Contact joelle.booth@plymouth.ac.uk to get started.

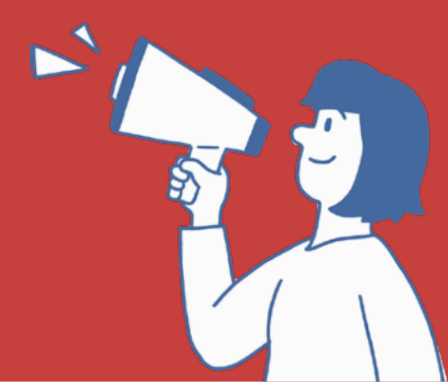

Faculty of Dental Surgery — Royal College of Surgeons of England

Peninsula Dental Social Enterprise

Southwest Inclusion Oral Health Research Group

University of Plymouth
